# The Role of Ten-Eleven Translocation Proteins in Inflammation

**DOI:** 10.3389/fimmu.2022.861351

**Published:** 2022-03-21

**Authors:** Christian Gerecke, Caue Egea Rodrigues, Thomas Homann, Burkhard Kleuser

**Affiliations:** Department of Pharmacology and Toxicology, Institute of Pharmacy, Freie Universität Berlin, Germany

**Keywords:** epigenetics, TETs, inflammation, DNA-methylation, DNA-hydroxymethylation, dioxygenases, immune cell regulation

## Abstract

Ten-eleven translocation proteins (TET1-3) are dioxygenases that oxidize 5-methyldeoxycytosine, thus taking part in passive and active demethylation. TETs have shown to be involved in immune cell development, affecting from self-renewal of stem cells and lineage commitment to terminal differentiation. In fact, dysfunction of TET proteins have been vastly associated with both myeloid and lymphoid leukemias. Recently, there has been accumulating evidence suggesting that TETs regulate immune cell function during innate and adaptive immune responses, thereby modulating inflammation. In this work, we pursue to review the current and recent evidence on the mechanistic aspects by which TETs regulate immune cell maturation and function. We will also discuss the complex interplay of TET expression and activity by several factors to modulate a multitude of inflammatory processes. Thus, modulating TET enzymes could be a novel pharmacological approach to target inflammation-related diseases and myeloid and lymphoid leukemias, when their activity is dysregulated.

## Introduction

Epigenetic modifications of DNA are essential for control of gene expression in cells. The 5´-methylation of cytosine in CpG-dinucleotides is one of the best-studied and most frequently observed epigenetic regulation element in mammalian cells. It plays a pivotal role in the establishment, maintenance, and persistence of gene expression patterns, contributing to nearly all cellular processes (1). Therefore, gene expression, which is modulated by epigenetic modifications, is placed by specific enzymes (“writers”), and recognized by effector proteins (“readers”). However, most of epigenetic marks are reversible, and various enzymes (“erasers”) remove these marks (2, 3). DNA methylation on the fifth carbon of cytosine in the CpG dinucleotide is carried out by “writer” DNA-methyltransferases (DNMTs), including DNMT1, DNMT3A, DNMT3B (whereas DNMT2 is considered a RNA-methyltransferase (4, 5), and is involved in different biological roles of various genomic regions (6). The DNA methyltransferases (DNMTs) convert cytidine to 5-methyl-2’-deoxycytidine (5-mdC) by transfer of a methyl group from S-adenyl methionine (SAM), which usually occurs on sites where a guanine nucleotide follows cytosine (CpG sites) in the DNA sequence (7). DNMT1 maintains the methylation pattern in DNA by binding to newly synthesized DNA and methylating it, copying the original methylation pattern. In contrast, DNMT3A and DNMT3B introduce methyl groups to DNA *de novo*. The maintenance of a balance between DNA hypomethylation and hypermethylation is crucial for physiological processes in the cell. Genome-wide DNA hypomethylation is associated with chromosomal instability, while loci-specific aberrant DNA hypermethylation in CpG islands of gene promoter regions can lead to gene silencing (8). This may result in facilitated tumorigenesis and cancer progression.

In the genome, 5-mdC are unequally distributed throughout the genome because the modified cytosine itself is mutagenic. It can undergo spontaneous hydrolytic deamination to cause C → T transitions, which in turn leads to severe DNA sequence changes (1). Nevertheless, approximately 70 - 80 % of CpGs are in a methylated status and therefore associated with transcriptional repression (9). However, in gene promoters CpGs are frequently unmethylated in order to control the transcriptional activity of the respective gene. Mostly, CpGs are clustered in CpG islands with a high content of cytosine and guanine base pairs (9). It has become clear that aberrant patterns of DNA methylation can lead to severe alterations in gene expression and be a fundamental part of carcinogenesis. Therefore, loss of normal DNA methylation occurs in DNA repetitive elements like silenced transposable elements (1). In contrast, aberrant DNA hypermethylation can be considered as a crucial event for the initiation and progression of cancer (10). This, in turn, can facilitate tumorigenesis and cancer progression due to decreased expression of e.g. tumor suppressor genes and disruption of cell cycle regulation, apoptosis, and/or DNA repair.

**Figure 1:**
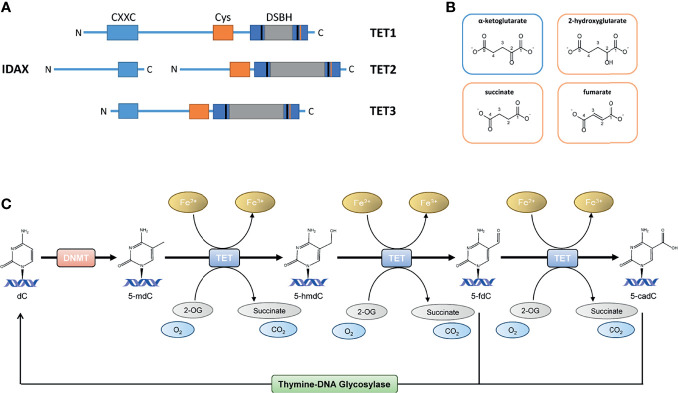
**(A)** The TET protein structures; full-length TET1 consists of 2039 aa, TET2 of 1921 aa and TET3 of 1803 aa. TET1, TET2 and TET3 have a C-terminal catalytic domain and contains a cysteine-rich domain and a double-stranded β-helix (DSBH) domain. A large low-complexity insert is found within the DSHB domain that may have regulatory roles *via* post-translational modifications. The N-terminal parts of TET1 and TET3 contain a DNA-binding CXXC domain. TET2 is lacking the CXXC domain, which CXXC finger protein 4 (IDAX) provides. **(B)** Structural similarities between the TET cosubstrate α-ketoglutarate (αKG) and the TET inhibiting molecules 2-hydroxyglutarate (2-HG), succinate and fumarate. **(C)** The TET enzyme family belongs to the αKG dependent dioxygenases (αKGD) superfamily. In mammals, TETs catalyze the oxidation of 5-methyl-2’-deoxycytidine (5-mdC) to 5-hydroxymethyl-2’-deoxycytidine (5-hmdC), 5−formyl-2’-deoxycytidine (5-fdC) and 5−carboxyl-2’-deoxycytidine (5-cadC), facilitating replication-independent DNA demethylation *via* thymine DNA glycosylase (TDG) base excision repair of 5-fdC and 5-cadC.

Once considered as non-reversible, recent technological advancements enabled great improvement in knowledge of DNA methylation dynamics and the possibilities of DNA demethylation processes (6). Although DNA demethylation *via* DNA replication-dependent passive dilution was known for several decades, the replication-independent active demethylation through the activity of the “eraser” enzymes ten-eleven translocation (TET) dioxygenases (TET1, TET2 and TET3) has just recently been described (11, 12). The TET enzyme family belongs to the α-ketoglutarate (αKG) dependent dioxygenases (αKGD) superfamily (synonymous to αKG is 2-oxoglutarate). In mammals, TETs catalyze the oxidation of 5-mdC to 5-hydroxymethyl-2’-deoxycytidine (5-hmdC), 5−formyl-2’-deoxycytidine (5-fdC) and 5−carboxyl-2’-deoxycytidine (5-cadC), facilitating replication-independent DNA demethylation *via* thymine DNA glycosylase (TDG) base excision repair of 5-fdC and 5-cadC (11, 12). The reaction mechanism is presented in **Figure 1**. However, more than a mere intermediate of DNA demethylation, 5-hmdC is a stable epigenetic modification distributed in a cell-, and tissue-type specific pattern and specially enriched at enhancers and gene bodies (13, 14). The TET-mediated conversion of 5-mdC to 5-hmdC at promoters, and subsequent DNA demethylation appears to be highly associated with transcriptional gene reactivation in several contexts (15, 16). As TET proteins and their products, i.e., oxidized cytosine bases, are involved in the maintenance of several biological processes, there has been accumulating evidence that they play a complex role in the regulation of inflammatory responses.

The TET enzymes are differentially expressed in several tissues during development and can regulate several conserved signaling pathways, which involve important transcription factors as Wingless (*WNT*), Notch, Sonic Hedgehog (*SHH*) and Transforming Growth Factor Beta (*TGFB*). Thus, the normal function of TET enzymes is fundamental for normal embryonic development and TET deficiency in animal models has shown to delay cell differentiation and result in dysregulated expression of genes involved in these signaling pathways. Consequently, the absence of TETs results in central nervous system defects and retinal deformity (17–22). Furthermore, TET deficiency plays a pivotal role in the initiation and progression of several malignancies and other aberrations.

In this review, we focus on highlighting the current understanding and emerging concepts in the mechanisms through which TET proteins and their products modulate inflammation in immune and non-immune cells, including also relevant aspects of the regulation of myeloid and lymphoid immune cell development, differentiation, and function. Finally, we present future potential perspectives of how these findings could pave the way to prevent and treat inflammation-related diseases. We outline current understanding of the roles of TET proteins in regulating adaptive and innate immune system and the crucial role in epigenetic modulation by promoting DNA demethylation, and producing oxidized products that affects these cells lineage and function. Additionally, we summarize how aberrant DNA-methylation plays a key role in proper hematopoietic stem and progenitor cells (HSPC) self-renewal and lineage differentiation dysregulation and can lead to aberrant stem cell function and cellular transformation.

## Mode of Action of TET Enzymes in Oxidation of 5-Methyl-2’-Deoxycytidine

**Figure 2:**
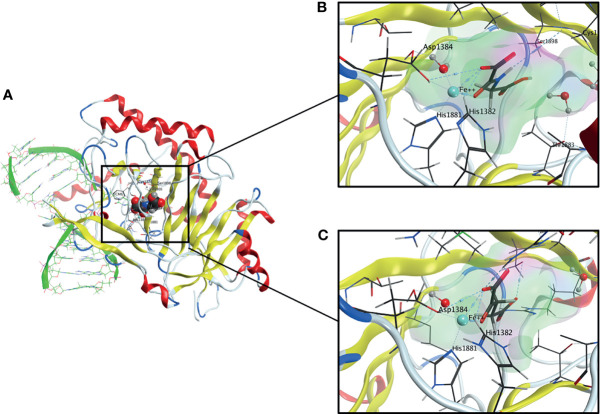
**(A)** Overall Structure of TET2-DNA Complex generated from pdb: 4NM6. The active site contains a highly conserved 2-His-1-carboxylate-amino acid residue triad motif in which the catalytically essential Fe(II) is fixated by two histidine residues and an aspartic acid residue. A water molecule is also an essential part of the complex. **(B)** The active site of TET2 containing N-Oxalylglycine (NOG). **(C)** The active site of TET2 containing the cosubstrate α-ketoglutarate (αKG).

Members of TET dioxygenase family are capable of oxidizing methylated 2’-deoxycytidines to 5-hmdC, 5-fdC and 5-cadC, which are eventually replaced by unmodified 2’-deoxycytidines because of TDG-mediated base excision repair (23–25). TET proteins belong to αKGD, employing Fe(II) as metal cofactor and αKG as cosubstrate (26, 27). The αKGD family has approximately 70 members in mammals. In addition to TETs, there exist numerous histone lysine demethylases, prolyl 4-hydroxylases that modify the hypoxia-inducible factor (HIF-P4Hs) or collagens (collagen P4Hs), the hypoxia-inducible factor asparagine hydroxylase FIH (factor inhibiting HIF) and FTO (fat mass and obesity-associated protein), the first identified RNA demethylase (15). The αKGDs, including TET enzymes, share the same reaction mechanism and cofactors, but their substrates vary from DNA to RNA, proteins and fatty acids. Besides Fe(II) and αKG, αKGDs require molecular oxygen (28, 29). The cofactors are coordinated at the active site by conserved residues, Fe(II) by two histidines and an aspartate and αKG by a positively charged arginine in TETs (**Figure 2**). The catalytic domains possess a double-stranded β-helix structure known as a jellyroll. Following cofactor and substrate binding, the molecular oxygen oxidizes Fe(II), inducing substrate oxidation and decarboxylation of succinate and CO_2_ (**Figure 1C**). The human TETs are large proteins, full-length TET1 being composed of 2039 amino acids (aa), TET2 of 1921 aa and TET3 of 1803 aa (23). The aminoterminal parts of TET1 and TET3 contain a DNA-binding CXXC domain (23), whereas this is lacking in TET2 but mediated by CXXC finger protein 4 (IDAX) (30). The catalytic domains are in the C-termini and contain a cysteine-rich domain and a double-stranded β-helix domain (**Figure 1A**). A large low-complexity insert is found within the double-stranded β-helix domain that may have regulatory roles *via* post-translational modifications. The human *TETs* are widely expressed, while experimental data suggest that *TET1* is preferentially expressed in embryonic stem cells (ESCs) whereas *TET2* and *TET3* are expressed in many tissues and have overlapping expression profiles (23). The catalytic activity of the TETs is strongly dependent on Fe(II) and αKG (15, 29). From a therapeutic point of view, a limited number of known TET enzyme activators can significantly increase genome-wide 5-hmdC levels. Vitamin C is one of the best-known substrates of the TET enzymes as well as a potent antioxidant and reducing agent (31). It has been proposed that vitamin C is responsible for the restoration of TET enzyme catalytic activity through the reduction of Fe(III) to Fe(II) (32, 33). This hypothesis has, however, been contradicted by other studies in which other strong reducing agents were unable to enhance the TET-mediated hydroxylation of 5-mdC (34–36). The mechanism by which vitamin C contributes to increased levels of TET-mediated hydroxylation of 5-mdC is, therefore, presently unclear. Additionally, retinol is proved to increase *TET2* expression in naïve ESCs (37). Besides, TET enzymatic activity is strongly dependent on the availability of reaction cofactors, including Fe(II), O_2_ and αKG (15). However, an activity suppression can occur *via* product inhibition of the αKG-metabolite succinate (**Figure 1B**). In this context, it is of great interest that although the oncometabolite 2-hydroxyglutarate (2-HG) has structural similarities to αKG, unlike this natural substrate, it inhibits TET enzymatic activity (**Figure 1B**) (15, 38–42). Indeed, the oncometabolite 2-HG is produced due to mutations of the isocitrate dehydrogenases genes *IDH1* and *IDH2*, which play a central role in the citric cycle. The resulted dysfunctional enzymes exhibit a neomorphic gain of function, which leads to a decreased amount of the essential TET cosubstrate αKG as it is irreversibly converted to the oncometabolite 2-HG. Certainly, a specific CpG island methylator phenotype in gliomas and colorectal cancers that is characterized by DNA hypermethylation has been associated with *IDH* mutations, which has led to several speculations that *IDH* mutations contribute to tumorigenesis *via* altered epigenetic regulation (43–45).

## The Role of Dysfunctional TET Enzymatic Activity in Development of Malignancies

**Figure 3:**
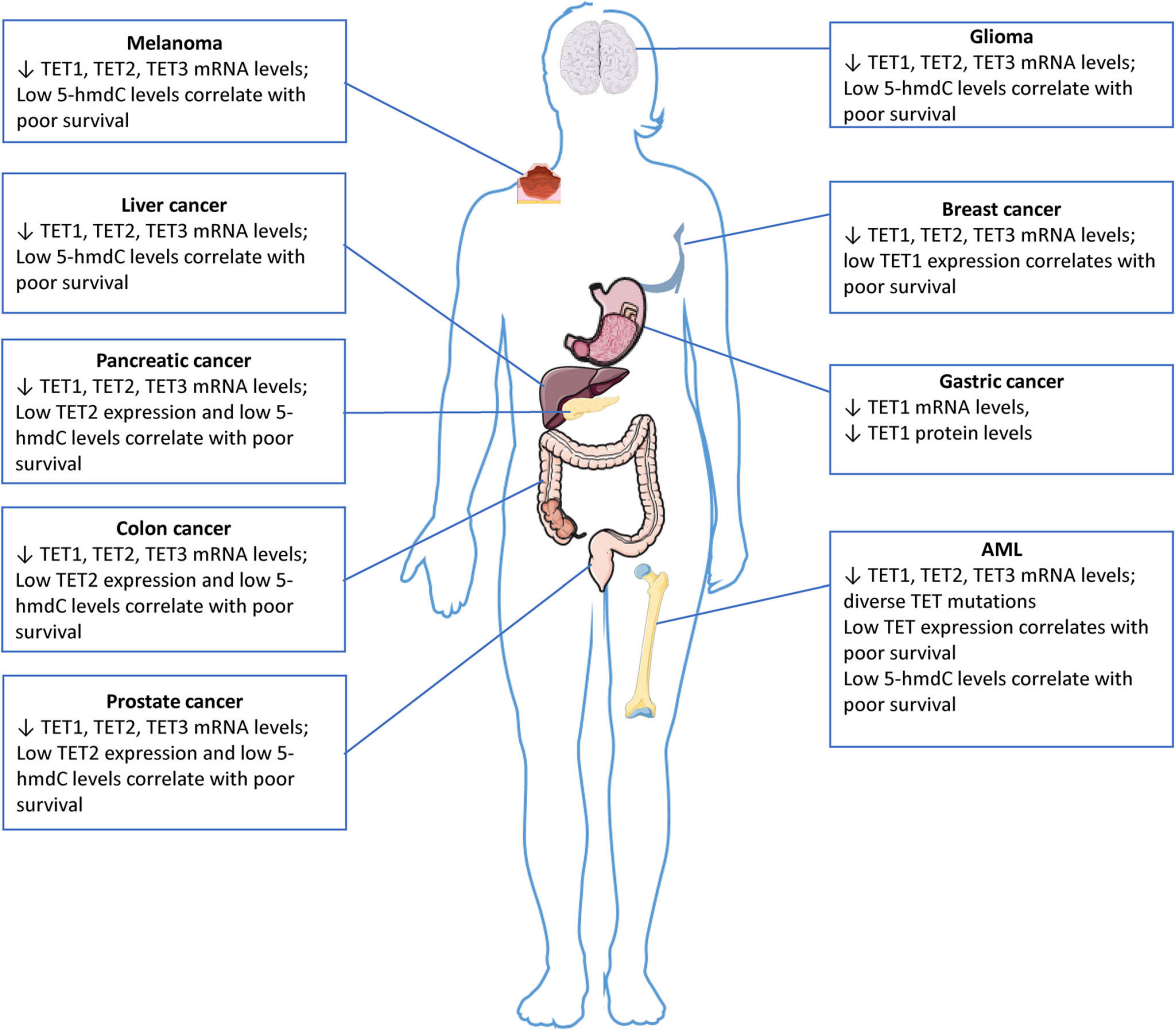
5-hmdC levels and TET expression in solid tumors and leukemia. The downregulation of TET gene expression, which is often associated with reduced 5-hmC levels, has been observed in numerous solid cancers. TET genes are rarely mutated in solid tumors, the impairment of their expression and activity as defined by reduced 5-hmC levels may be caused by other factors. The images were provided and adapted from Servier Medical Art (smart.servier.com).

Aberrant DNA hypermethylation has been associated with several types of cancer, including gliomas, acute myeloid leukemia (AML) and cancers of the lung, breast, ovaries, and colon (46–48). Additionally, a genome-wide decrease in 5-hmdC levels is considered an epigenetic hallmark in many cancers (29, 49) and several studies have highlighted the diagnostic and prognostic value of this mechanism. In hematological malignancies, *TET1* was first identified as a gene fused to *MLL* (mixed-lineage leukemia) in an AML patient with a ten–eleven translocation (50); however, this translocation does not occur very frequently (51). Loss of function mutations in genes encoding TET enzymes occur frequently in hematopoietic malignancies, but rarely in solid tumors, which instead commonly have reduced enzymatic activity (**Figure 3**). The impairment of their expression and activity as defined by reduced 5-hmdC levels may be caused by other factors. Thus, in glioma, *TET2* mutations have not been described; however, *TET2* promoter methylation has been detected in 14% of low-grade glioma patients without *IDH* mutation (52, 53). Since promoter methylation is associated with transcriptional repression, this suggests a decreased *TET2* expression in these patients, possibly leading to decreased levels of 5-hmdC. In addition, Müller et al. (54) showed a strong correlation between nuclear exclusion of TET1 and decreased levels in glioma. In their study, 61% of the tumor samples had decreased 5-hmdC levels. Of the 5-hmdC negative tumors, 70% showed nuclear exclusion of TET1, or no detectable TET1 protein, thereby demonstrating an additional mechanism that may lead to decreased 5-hmdC in glioma.

TET proteins may also be post-transcriptionally down-regulated by microRNAs (miRNA) (55, 56). The miRNA miR-29 inhibits the translation of *TET1* and *TDG* in lung cancer cells, in human dermal fibroblasts and vascular smooth muscle cells miR-29 inhibits formation of all TET isoforms resulting in effective 5-hmdC decrease (57, 58). Moreover, miR-26 and TET or TDG levels are inversely correlated during pancreatic progenitor cell differentiation (59). Additionally, in breast cancer the TET family is a target of miR-22. Here, it promotes invasiveness and metastasis by DNA methylation-dependent silencing of miR-200 through the direct targeting of TET proteins (56). Moreover, miR-22 is overexpressed and *TET2* and *miR-200* are downregulated in patients with gastric cancer, and the aberrant expression of both correlates with poor survival (60). Interestingly, in embryonal kidney cells the ancestor of the CXXC domain for *TET2*, IDAX, may stimulate caspase-dependent TET2 cleavage after targeting it to DNA (30). The interaction of TET proteins with OGT (O^6^-Guanine transferase) enables catalytic-independent gene expression regulation (61). Due to the common alteration of OGT in many cancers, it may affect the TET activity on oxidizing DNA, as well (62). Additionally, various TET-interacting proteins, such as EZH2 (Enhancer of zeste homolog 2), SIN3A (SIN3 transcription regulator family member A), the hematopoietic transcription factor SPI1 and EBF1 (B cell factor 1), may modify the actions of TET proteins; SPI1 (involved in the differentiation of B cells and myeloid lineages) is often deregulated in leukemia and potentially influences mRNA splicing (63). Another study found that TET2 mediates the IFN-γ/JAK/STAT signaling pathway to control chemokine and PD-L1 (Programmed cell death-ligand 1) expression, lymphocyte infiltration, and cancer immunity (64). Generally, reduced TET activity was associated with decreased Th1-type chemokines and tumor-infiltrating lymphocytes and the progression of human colon cancer. Deletion of *TET2* in murine melanoma and colon tumor cells reduced chemokine expression and tumor-infiltrating lymphocytes, enabling tumors to evade antitumor immunity and to resist anti–PD-L1 therapy. Conversely, systematic injection of the TET activating compound vitamin C increased chemokines and tumor-infiltrating lymphocytes, leading to enhanced antitumor immunity and anti–PD-L1 efficacy and extended lifespan of tumor-bearing mice. It has been suggested, that TET activity could serve as a biomarker for predicting the efficacy and patient response to anti–PD-1/PD-L1 therapy. Additionally, stimulation of TET activity could serve as an adjuvant immunotherapy of solid tumors.

Especially hematological malignancies are often linked to loss of function mutations in *TET* genes (65), which leads to impaired cell differentiation and transformation (66, 67). Furthermore, four genes that play a role in the citric acid cycle, namely *IDH1, IDH2, SDH* (succinate dehydrogenase) and *FH* (fumarate hydratase), are frequently mutated in leukemias and various types of solid cancers (38, 68, 69). These genes are able to affect the activity of the TET proteins by changing the levels of metabolites that compete with the TET cofactor αKG. In humans, *TET2* is one of the most frequently mutated genes in hematopoietic cancers of both myeloid and lymphoid origin (66). Human and murine hematopoietic progenitor and mature immune cells show a high *TET2* expression and high levels of 5-hmdC (70). *TET2* has been extensively shown to play an important role in the regulation of hematopoietic stem cell expansion, differentiation, and function (71). Loss of function *TET2* mutations are often described as the first step in the multi-hit model of development of leukemia (72). Even before the discovery of the TET enzymatic function, deletions and somatic mutations in *TET2* were identified across multiple exons in different types of blood cancers in humans (72). In myeloid leukemias, e.g., in ∼30% of cases of secondary AML, ∼17% of *de novo* AML, ∼30% of myelodysplastic syndromes (MDS), ∼50% of chronic myelomonocytic leukemias (CMMLs), and ∼20% of myeloproliferative neoplasms (MNP) show a mutated *TET2*. Additionally, *TET2* is also found to be mutated in certain lymphoid leukemias, e.g., ∼17% of T-cell acute lymphoblastic leukemia (T-ALL), and in certain lymphomas, e.g., ∼33% of angioimmunoblastic T lymphomas, and in ∼12% of diffuse large B-cell lymphomas (73–75). *TET2* mutations are shown to increase with ageing, being observed in peripheral blood cells of ∼5% - 10% of adults older than 65 years of age, and are one of the most commonly detected alterations in clonal hematopoiesis of indeterminate potential (CHIP). CHIP is a result from *TET2*-mutant hematopoietic stem cell proliferative advantage and is considered a preleukemic condition that accounts as a potential driver of myeloid dysfunction and development of inflammatory diseases such as type 2 diabetes, coronary heart disease, and ischemic stroke (76). Data from a recent meta-analysis has shown that in spite of the fact that *TET2* mutations had no significant prognostic value on myelodysplastic syndromes, the response rates to hypomethylating agents were significantly different between patients with and without *TET2* mutations (77). Both *TET2*-knockout murine and *TET2*-mutant human hematopoietic stem cells display resistance to colony-suppressive effects of TNF-α, therefore sustaining proliferative advantage in an inflammatory environment (78). In summary, the role of TET enzymes in the development of cancer has been examined and extensively proven (16, 66, 79) (**Figure 3**).

## The Role of TET Enzymes in Myeloid Cell Lineage Differentiation and Function

**Figure 4:**
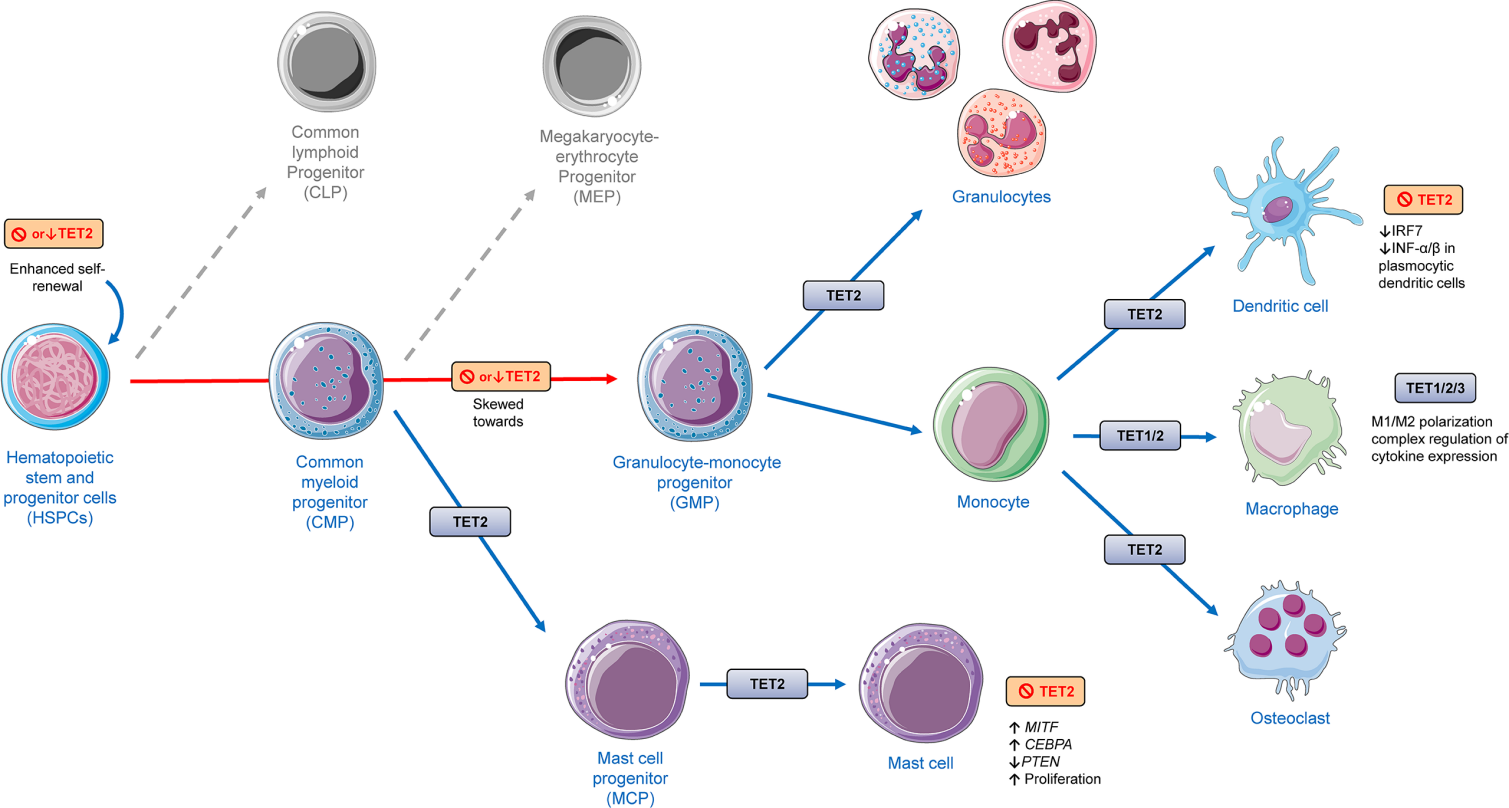
The role of TET proteins in myeloid cell lineage differentiation and function based on findings from different models presented in this review, including human and murine cells and animal experiments applying rodents. TET2 deletion has shown to promote increased hematopoietic stem and progenitor cells (HSPCs). TET2 has shown to be involved in different aspects of the differentiation of myeloid cells. Deletion or depletion of TET2 was shown to skew differentiation of HSPCs towards monocytic/granulocytic lineages. TET2 regulates the function of monocytic populations such as dendritic cells, macrophages, and osteoclasts. TET1/2 have been shown to play a role in monocyte to macrophage differentiation. All TET members (TET1/2/3) have been shown to have an effect in the function of macrophages, affecting their activation, polarization (M1/M2) and cytokine expression. TET2 was shown to regulate mast cell differentiation and function in both a catalytic-dependent and -independent manner. The cellular images were provided and adapted from Servier Medical Art (smart.servier.com).

The cells of the innate immune system are often recognized as the first line of host defense against pathogens and injury, being fundamental not only for the initiation but also for the resolution of inflammatory processes. Here, myeloid cells, including macrophages, dendritic cells, neutrophils, mast cells and, with the involvement of epithelial and endothelial cells as well, are identified as important players in the recognition and response to potentially harmful external and internal stimuli. This system recognizes pathogens *via* a limited number of germline-encoded pattern-recognition receptors (PRRs), including toll-like receptors (TLRs), RIG-I-like receptors (RLRs) and the NOD-like receptors (NLRs), which initiate complex intracellular responses upon stimulation (80–85). All TET proteins (TET1-3) have been implicated in the differentiation and/or function of myeloid cells, regulating key aspects related to inflammation, which is illustrated in **Figure 4**.

TET1 has been suggested to play a role in the differentiation from monocytes to macrophages and to the activation of proinflammatory phenotype of macrophages. In the human monocytic leukemia cell line THP-1, *TET1* mRNA is increased during macrophage differentiation induced by macrophage colony-stimulating factor (M-CSF). Moreover, 5-hmdC levels were shown to be enhanced on global levels and specifically in the *TNF-α* promoter during the differentiation of monocytes to macrophages. Importantly, CRISPR stable knockout of *TET1* led to decreased expression of *TNF-α* and other pro-inflammatory cytokine genes, suggesting that TET1 is an important activator of the *TNF-α* gene in macrophages (86). In line with these findings, *TET1* knockdown leads to inhibition of M1 macrophage polarization through the NF-κB (nuclear factor kappa B) signaling pathway. LPS/IFN-γ-stimulated macrophages were shown to display significant decrease in the production of proinflammatory markers such as IL-6, TNF-α, CCL2, and HLA-DR, when *TET1* was down regulated (87). In a recently published study, TET1 has been shown to display a striking specificity in the regulation of gene expression in macrophages related to cell migration and trafficking. In LPS-stimulated macrophages, *TET1* deletion repressed the expression of *CXCL1* (C-X-C Motif Chemokine Ligand 1), a chemokine that regulates cell migration and recruitment, playing a role in neutrophil influx during lung inflammation, for example (88).

In primary murine bone-marrow-derived macrophages, TET3 was shown to inhibit type I IFN production after poly(I:C) stimulation or viral infection (89). Thus, this study showed that deletion of *TET3* in macrophages elicited enhanced antiviral responses. Mechanistically, TET3 suppressed IFN-β production through the recruitment of the histone deacetylase 1 (HDAC1) to the *IFNB1* promoter, a mechanism that is independent from its 5-mdC oxidative catalytic activity (89). Non-classical monocytes comprise around 2 - 11% of circulating monocytes and have distinguished expression of surface markers, transcriptomic and metabolic profiles in comparison to classical monocytes with complex functions in homeostasis control and in the pathophysiology of chronic inflammatory diseases (90, 91). Recently, TET3 has been shown to be involved in the control on the regulation of the repartition between subsets of classical and non-classical monocytes. In fact, *TET3* knockout mice were shown to display an increased number of non-classical monocytes. In this context, it is of interest that non-classical monocyte numbers are significantly decreased in CMML patients (92).

There is an accumulating body of evidence that loss or depletion of *TET2* leads to alterations in differentiation and function of different types of cells of myeloid lineage and their progenitors. Short hairpin RNA (shRNA)-mediated depletion of *TET2* in murine bone marrow HSPCs has shown to skew their differentiation towards monocyte/macrophage lineages (93). Similarly, results from another working group shown that their *TET2*-knockout generated mouse model displayed increased Lin^−^Sca-1^+^c-Kit^+^ (LSK) cell pool with increased hematopoietic repopulating capacity and altered cell differentiation skewing toward monocytic/granulocytic lineages in an *in vitro* competitive reconstitution assay (94). Evidence from *in vivo* and *in vitro* experiments suggest that although *TET2* loss does not affect markers of terminal macrophage differentiation, *TET2* plays an important role in macrophage functional polarization (95). *TET2* gene expression in murine macrophages has been shown to be induced by LPS treatment, an effect that was abolished by pretreatment with BAY 11–7082, an NF-κB inhibitor. Interestingly, here it was shown that unstimulated *TET2* ^–/–^ murine macrophages displayed increased gene expression of multiple proinflammatory cytokines and chemokines, while LPS-stimulated *TET2* ^–/–^ macrophages demonstrated impaired ability to resolve inflammation, expressing increased mRNA levels of *IL1B*, *IL6*, and *ARG1* (*Arginase 1*) at later stages of LPS stimulation (95). Similar findings were observed with murine and human *TET2* ^–/–^ dendritic cells and macrophages. Furthermore, animal experiments demonstrated that *TET2*-deficient mice were more susceptible to colitis and to endotoxin shock in comparison to wild-type mice, displaying a more severe inflammatory phenotype and severe tissue damage (96). Mechanistic investigation revealed that *TET2* interacts with IκBζ, a specific transcription factor for *IL6*, to promote *IL6* transcription repression during inflammation resolution through the subsequent recruitment of HDAC2 (Histone Deacetylase 2) to the *IL6* promoter, a mechanism that is independent from TET2 catalytic activity (96).

Evidence on the transcription repression of *IL1B* in macrophages mediated by *TET2 via* HDAC–mediated histone deacetylation of *IL1B* promoter has also been shown. Beyond its effect on the transcription of *IL1B*, *TET2* deficiency in LPS/IFN-γ/ATP–treated macrophages was further shown to enhance the priming of NLRP3 inflammasome, promoting increased cleavage of pro–IL-1B to its active form. Moreover, results from animal experiments from this working group have revealed that *TET2* loss of function in macrophages led to increased proatherogenic activity, suggesting a pivotal role of *TET2*-deficient macrophages in the acceleration of atherosclerosis that is associated with expansion of *TET2*-deficient HSPCs (97). In another study, *TET2*-deficiency in hematopoietic cells by both, specific ablation in myeloid cells and by partial reconstitution of the bone marrow with *TET2*-deficient HSPCs, led to exacerbated IL-1B, resulting in cardiac dysfunction with worse late-stage cardiac remodeling in two experimental murine models of heart failure (98). Consistently, findings from both studies have shown that the treatment with MCC950, a specific NLRP3 inflammasome inhibitor, was able abrogate the exacerbated IL-1B secretion, promoting an atheroprotective effect and hindering the development of heart failure in these murine models for clonal hematopoiesis associated with *TET2* somatic mutations (97, 98).

Plasmacytoid dendritic cells express endosomal sensors TLR7/9 and have a massive capacity of producing INF, playing a fundamental role in both innate and adaptive immune responses against DNA and RNA viral infections. In order to elicit INF-α/β production, these cells activate upon stimulation important transcription factors such as IRF7 (interferon regulatory factor 7), NFκB and AP-1. TET2 was shown to be recruited by the zinc finger CXXC family epigenetic regulator CXXC5 to the CpG island containing promoter of *IRF7*, regulating its hypomethylation and thereby increasing its gene expression. In comparison to controls, *TET* deficient dendritic cells displayed decreased capacity to express *IRF7*. These findings suggest that along with CXXC5, TET2 is an important proinflammatory antiviral defense regulator of dendritic cells (99).

Recently, *TET2* has also shown to be post-transcriptionally regulated during inflammatory processes affecting cytokine expression through macrophage activation *via* feedback regulatory mechanisms mediated by miRNAs (100, 101). Using different knockout and myeloid cell-specific transgenic mouse models, it has been shown that in LPS-activated bone-marrow derived macrophages, the miR-let-7a/let-7d/let-7f cluster (*let-7adf*) promotes IL-6 secretion by two different mechanisms. In the direct mechanism, let-7adf targets specifically *TET2*, thereby decreasing its expression at mRNA and protein levels. Indirectly, let-7adf has been suggested to regulate the Lin28a/SDHA axis in these LPS-activated macrophages (100). This miRNA cluster directly degradates *TET2* mRNA and increases succinate levels, which inhibits TET enzymatic activity, as described above (102, 103).

Further, miR-125a-5p expression is upregulated in vascular endothelial cells treated with oxidized low-density lipoprotein. It targets the *TET2* 3′-untranslated region and so downregulates *TET2* expression in a concentration-dependent manner. In direct consequence, these cells displayed decreased levels of 5-hmdC, mitochondrial dysfunction, increased reactive oxygen species, activation of NF-κB, NLRP3 inflammasome activation, increased release of IL-1B and IL-18 and cell death through pyroptosis (101). Interestingly, miR-125a-5p expression is enhanced in LPS-stimulated murine bone marrow-derived macrophages, suppressing the expression of the M1 phenotype while promoting the expression of the M2 phenotype (104). In contrast, another study has shown that LPS/IFN-γ-stimulated tumour-associated macrophages with increased expression of miR-125a-5p led to promotion of M1 polarization by targeting the factor FIH1 (inhibiting hypoxia-inducible factor-1 α) and inhibition of M2 polarization through targeting interferon regulatory factor 4 (*IRF4*) (105). Due to these contradictory results, further research needs to unveil possible mechanistic links between miR-125a-5p and TET2 to coordinate macrophage polarization.

*TET2* has also shown to regulate mast cell differentiation, proliferation, and function (106–108) (**Figure 4**). *TET2* loss of function in mast cells affects the 5-hmdC deposition and alters cytokine production. Cell culture experiments have shown that *TET2* ^−/−^ murine bone marrow progenitor cells displayed delayed differentiation to mast cells, while *TET2* ^−/−^ mast cells displayed altered 5-hmdC patterns, disrupted gene expression, decreased percentage of cells expressing IL-6, TNF-α, and IL-13 upon IgE and antigen stimulation, and marked increased proliferation. Interestingly, although vitamin C was able to enhance 5-hmdC levels and partially rescue the differential gene expression in *TET2* ^−/−^ mast cells, suggesting existing compensatory mechanisms, proliferation differences between *TET2* ^−/−^ and *TET2* ^+/+^ cells remained unaffected (107). In another study, in comparison to controls, *TET2* ^–/–^ mice display increased numbers of immature promastocytes in the peritoneal cavity. RNA-sequencing analysis on bone marrow-derived mast cells derived from *TET2* ^–/–^ mice revealed transcriptional repression of genes required for mast cell differentiation, maturation, and function. *TET2* ^–/–^ mast cells displayed marked upregulation of genes involved in in inflammatory response, migration, growth, proliferation, and antiapoptotic mechanisms, whereas genes involved in negative regulation of cell proliferation and phosphorylation were downregulated. Importantly, *TET2* ^–/–^ mast cells display dysregulated expression of transcription factors, including *MITF* (Melanocyte Inducing Transcription Factor) and CEBPα (CCAAT/enhancer-binding protein alpha), and show reduced *PTEN* (Phosphatase and tensin homolog) expression because of *PTEN* promoter hypermethylation and hyperactivation of the PI3K/AKT/c-Myc pathway. Interestingly, *PTEN* expression and maturation was rescued in *TET2^–/–^* mast cells treated with the FDA-approved hypomethylating agent 5-azacytidine, whereas the co-treatment of these cells with 5-azacytidine and vitamin C led to a complete restoration of mast cell maturation and correction of hyperproliferation (106).

In mRNA oxidation-dependent manner, TET2 was shown to promote pathogen infection-induced myelopoiesis through *ADAR1* (Adenosine deaminase acting on RNA)-mediated repression of *SOCS3* (suppressor of cytokine signaling) expression at the post-transcription level. In mice experiments, TET2 regulated both, abdominal sepsis-induced emergency myelopoiesis and parasite-induced mast cell expansion. In comparison to controls, a *TET2*-deficient mice model of abdominal sepsis with acute mobilization and expansion of myeloid cells have shown to be protected from sepsis with lower mortality rates. Interestingly, while control mice developed neutrophilia and inflammatory monocytosis with increased serum levels of inflammatory cytokines (e.g., TNF-α, keratinocyte chemoattractant, macrophage inflammatory protein-1α), neutrophil and monocyte numbers were barely altered in *TET2*-deficient mice (108).

## The Role of TET Proteins in B and Plasma Cell Differentiation and Function

**Figure 5:**
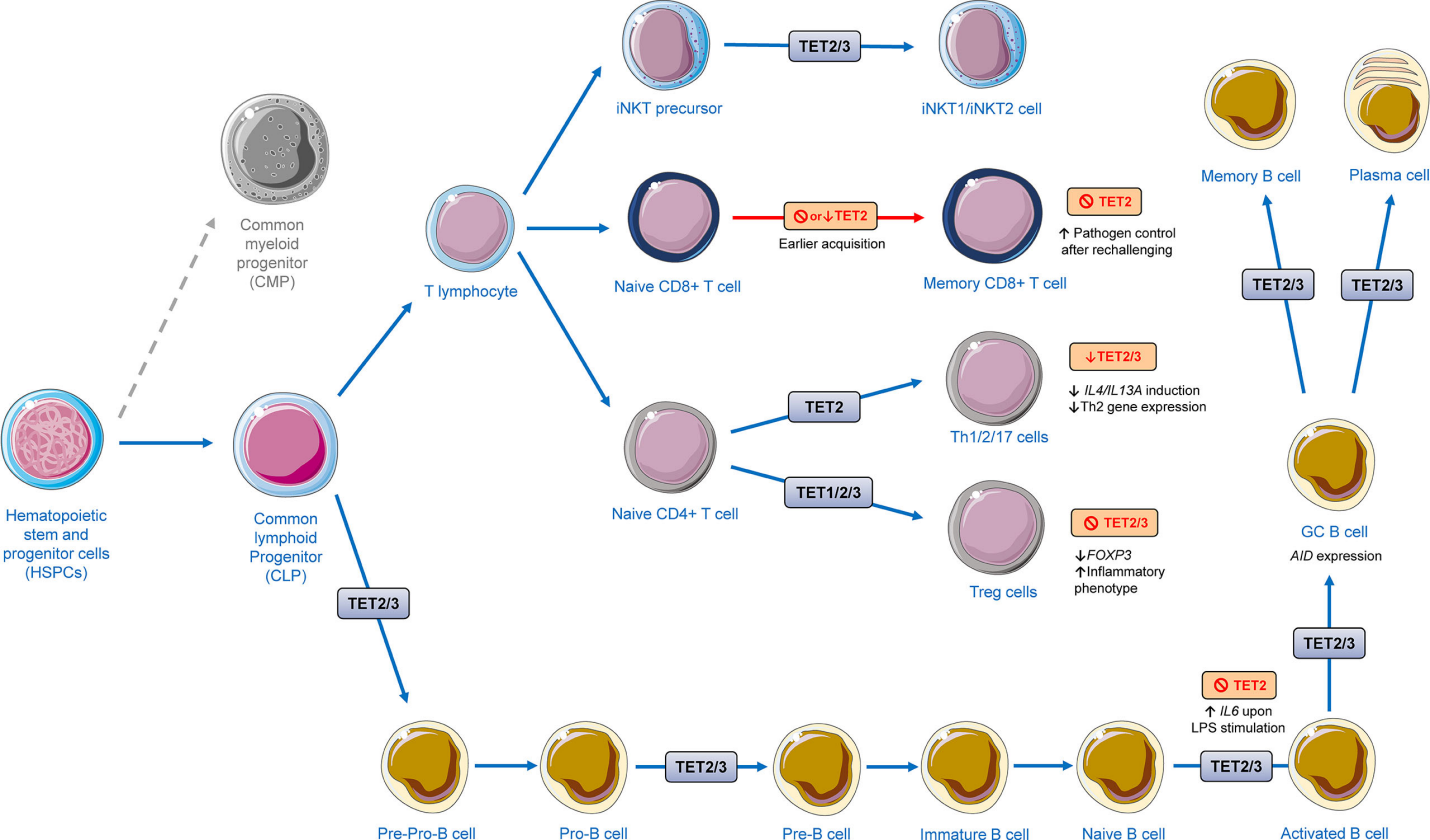
Regulation of lymphoid development and function by TET proteins. The presented scheme summarizes data from *in vitro* and murine models highlighted in this review. TET proteins have a great impact on the development of different lymphoid cells. TET2/3 were shown to regulate invariant natural killer T (iNKT) cells maturation and development and differentiation into iNKT1 and iNKT2. Ablation or depletion of TET2 has been shown to lead to early acquisition of memory CD8+ T cell without disrupting effector function after acute viral infection. TET2 has shown to play a role in differentiation of T helper (Th) cells. Knockdown of TET2/3 gives rise to the decreased responses of IL-4/13A induction against exogenous soluble antigen stimulation, leading to a restrained expression of Th2-related genes. TET1/2/3 regulate the stability of the regulatory T cells (Treg cells). TET2/3 deletion leads to decreased *FOXP3* expression and a shift to an inflammatory phenotype. During B cell differentiation, TET2 and TET3 orchestrate B cell maturation and function. The cellular images were provided and adapted from Servier Medical Art (smart.servier.com).

B cells and plasma cells belong classically to cells of the adaptative immune system and play an important role in acute and chronic inflammatory processes, being involved in pathophysiological mechanisms of several inflammatory and autoimmune diseases due to their ability to drive or suppress inflammation (109–111). TET enzymes affect different aspects of B cell development, differentiation, and function, which is presented in **Figure 5**. Recent sequencing approaches of different human B cell subpopulations revealed intense alterations of the epigenomic landscape during B cell differentiation, characterized by progressive demethylation resulting in hypomethylation at later stages (112). While *TET1* has been identified as a tumor suppressor of B cell lymphoma and its loss in HSPCs promotes differentiation bias towards B cell lymphopoiesis, its expression was shown to be decreased during B lineage commitment (113). The mRNA levels of *TET2* and *TET3*, on the other hand, were observed to progressively increase during B cell development (114). In mice, *TET2* and *TET3* conditional knockout at early stages of B cell development has been shown to largely impede lineage-specific programmed demethylation events (115). Interestingly, *TET2* and *TET3* were shown to cooperate with B linage-specific transcription factors to promote 5-mdC oxidation and demethylation, thereby improving chromatin accessibility at B cell enhancers. Loss of both, *TET2* and *TET3* in mice has shown to lead to blockade of pro- to pre-B cell transition in the bone marrow, decreased *IRF4* expression at mRNA and protein levels and impaired the germline transcription and rearrangement of the Ig light chain (Igκ) locus (116).

Antigenic activation of B cells results in the formation of dynamic germinal centers (GC). Here, B cells clonally expand and undergo activation induced cytidine deaminase (AID)-induced hypermutations that are essential for shaping antibody affinity and diversity. Upon GC exit, B cells differentiate into antibody-secreting plasma cells and memory B cells (117). Both *TET2* and *TET3* have shown to play a role in antibody class switch recombination (118). In mice experiments, *TET2* deletion alone was enough to disrupt transit of B cells through GCs, leading to GC hyperplasia, impairment of class switch recombination, and blockade of plasma cell differentiation. Remarkably, in a cohort of 128 patients with diffuse large B-cell lymphomas, *TET2* loss of function mutations was found in ∼12% of the cases (119). In *TET2* knockout mice, AID-induced demethylation was impaired and an aberrant DNA hypermethylation of regulatory elements was observed with potential disturbance on the binding of important transcription factors, such as transcription factors involved in exit from the GC reaction and B cell receptor, antigen presentation, and CD40 pathways. Importantly, hypermethylation signatures of murine GC B cells were found to be significantly reflected in primary cells from *TET2* mutant patients with large B-cell lymphomas (120). It has been shown that TET is required for optimal AID expression, and both *TET2* and *TET3* were shown to guide the GC exit of B cells to antibody secreting plasma cells (114). Mechanistically, *TET2* and *TET3* enhance the expression of *AID* by depositing 5-hmdC and facilitating DNA demethylation at TET2-responsive enhancer elements located within the *AID* superenhancer, thereby maintaining chromatin accessibility and promoting gene expression (121).

*TET2*-deficient B cells were shown to express higher level of IL-6 but not TNF-α in response to LPS stimulation. These findings further demonstrate the general characteristics of *TET2* in repressing *IL6* transcription in B cells and indicate that *TET2* may exert its broad repression of proinflammatory genes in different types of immune cells (96). TET2 and TET3 have also been implicated in the regulation of key aspects to prevent autoimmune inflammatory processes. The expression of the co-stimulatory molecule CD86 was shown to be upregulated on *TET2*- and *TET3*-deficient B cells, which displayed a permissive chromatin state at the *CD86* locus as a result of decreased accumulation of HDACs. Dysregulated *CD86* expression plays a role to the induction of autoimmune inflammatory processes, contributing significantly to aberrant activation of T and B cells *in vitro* and *in vivo* (122). Further research of how TETs regulate inflammation and autoimmune-related processes in B and plasma cells might reveal novel potential pharmacological targets and strategies to modulate autoimmune and chronic inflammatory diseases.

## The Role of TET Proteins in T Cell Differentiation and Function

T cells, cells that are generally seem as members of the adaptive immune system, have shown to play a prominent role in acute and chronic inflammatory processes (123, 124). Genome-wide mapping of 5-hmdC levels in T cells has revealed dynamic changes during sequential steps of lineage commitment in the thymus and the periphery. During the process of T-cell development and differentiation, with 5-hmdC enrichment in gene bodies of certain genes of developing T cells being strongly positive correlated with respective gene expression (125). *TET2* and *TET3* are expressed at high levels in thymocytes and peripheral T cells, and are responsible for the majority of 5-hmdC modifications in these sets of T cells (**Figure 5**) (126–129). It has been shown, that the deletion of both, *TET2* and *TET3*, in murine T cells caused a massive lymphoproliferative phenotype with enlarged spleen and lymph nodes. The *TET2/TET3* double knockout mice did not live longer than 8 weeks due to decreased thymic cellularity, lower number of CD4^+^/CD8^+^ double positive cells, and an increased percentage of CD4^+^ and CD8^+^ single positive cells, including phenotypes reminiscent of thymic atrophy induced by stress or inflammation (130).

Depending on several signals, naïve CD4^+^ T cells can differentiate into multiple lineages, including T helper cells like, Th1, Th2, Th17, follicular T helper cells (Tfh), and regulatory T cells (Treg). These differentiated cells are characterized into these five major subsets based on their expression of signature cytokines and lineage-specific master transcription factors and play a critical role in coordinating actions of other immune and non-immune cells during inflammatory processes (131). CD4^+^ T cells homeostasis is important for healthy immune responses, but also plays a role in the pathogenesis of immune-mediated inflammatory diseases such as multiple sclerosis (MS), psoriasis, and asthma (132). *TET2* has been shown to be more expressed in TCR (T-cell receptor)-activated Th cell subsets in comparison to *TET1* and *TET3*. TET2 recruitment to the signature cytokine loci was dependent on lineage-specific transcription factors, regulating differentiation of Th1 and Th17. Under Th1 cell polarization *in vitro*, *TET2^−/−^* T cells displayed a marked decreased expression of IFN-γ at mRNA and protein levels, whereas *TET2^−/−^* Th17 cells displayed a decrease in the expression of IL-17 on mRNA and protein levels in comparison to controls. Using a mice model of myelin oligodendrocyte glycoprotein peptide-induced experimental autoimmune encephalomyelitis, *TET2* expression has been shown to play a role in suppression of disease severity by regulating the expression of *IL10, IL17*, and *IFNG* in Th cell subsets (133). Importantly, *IL10* expression is considered to be generally an anti-inflammatory cytokine that plays a fundamental role in preventing inflammatory and autoimmune diseases (134).

Treg cells are known for their anti-inflammatory properties, promoting tissue repair, and suppressing effector T cell proliferation (135–138). Under specific conditions, however, these cells may lose suppressive properties, releasing proinflammatory cytokines and consequently contributing to inflammation-related diseases such as in certain autoimmune diseases (139). The transcription factor Forkhead box P3 (*FOXP3*) is crucial for Treg cells and its expression is imperative and sufficient for their suppressive activity. A decrease of *FOXP3* expression can shift the function of Treg cells, leading to expression of proinflammatory phenotypes with increased secretion IFN-γ and IL-17 (140). TET proteins have been shown to mediate the increase of 5-hmdC and loss of 5-mdC in Treg cell–specific hypomethylated regions, including conserved noncoding sequence elements (CNS) 1 and 2, intronic cis-regulatory elements in the *FOXP3* locus. Moreover, *TET2/TET3* deficient mice displayed markedly compromised stability of *FOXP3* expression. Vitamin C potentiates TET activity thereby promoting *FOXP3* expression stability (141). Treg cell specific demethylation region (TSDR) demethylation such as the demethylation of CNS2 has been shown to be required for a Treg immuno-suppressive phenotype induction in experiments that applied CRISPR-dCas9-TET1-mediated targeted DNA demethylation technology (142).. During thymic Treg development, IL-2 has been shown to be required to maintain increased expression levels of TET2, whereas TET2 downregulation was shown to prevent Treg cell specific demethylation region (TSDR) (143). In contrast, IL-6 has been shown to induce the expression of pro-inflammatory cytokines in Foxp3+ Treg cells. IL-6 signaling has been shown to induce Dnmt3a expression and activation, leading to DNA methylation of CNS2 and Foxp3 downregulation (144). Decreased TET2 and TET3 was shown to compromise physiological Treg function in animal experiments in which mice lacking *TET2* and *TET3* in Treg cells develop inflammatory disease with splenomegaly and leukocyte infiltration into the lung. Treg cells from these mice displayed dysregulation of Treg signature genes while also upregulating genes involved in cell cycle, DNA damage, and cancer, acquiring ultimately an inflammatory phenotype (145). Recently, miR142-3p has been shown to target *TET2*, impairing Treg cell differentiation and stability in models of type 1 diabetes, contributing to immune activation and progression during islet autoimmunity. Thus, inhibition of miR142-3p improved islet autoimmunity in animal experiments (146).

CD8^+^ T cells play an important role in the defense against pathogens and are also potentially important in defense against cancers (147). TET2 also regulates CD8^+^ T cell differentiation and function. Concomitant IL-12 and TCR-mediated stimulation of human naïve CD8^+^ T cells leads to TET2-mediated demethylation of the *IFNG-* promoter eliciting an increase in IFN-γ expression (148). Interestingly, in a murine model of acute viral infection, *TET2* loss led to early acquisition of a memory CD8^+^ T cells without disrupting antigen-driven cell expansion or effector function after acute viral infection, with *TET2*-deficient memory CD8^+^ T cells displaying superior pathogen control after rechallenging. *TET2* loss in these cells elicited altered DNA methylation patterns with the majority of differently methylated regions located in introns and coding sequences of genes involved in cellular growth, proliferation, development, death, and survival (149). Furthermore, concomitant deletion of *TET2* and *TET3* in mouse CD4^+^/CD8^+^ double-positive thymocytes have also shown to dysregulate development and proliferation of invariant natural killer T cells, suggesting that TET2 and TET3 carry proper development and maturation of these cells by suppressing aberrant proliferation mediated by TCR (130).

Altogether, there is data that demonstrates that TETs play an important role in different subset populations of T lymphocytes, affecting their differentiation and function. Future target research to understand mechanisms by which TETs regulate function of these cells during inflammatory processes in inflammation-related diseases might lead to the finding of new venues for inflammation-related disease prevention and treatment.

## The Involvement of TETs in the Regulation of Neuroinflammation

Neuroinflammation is an important defense mechanism of the central nervous system against pathogenic and or infectious insults that has also been identified as a common etiopathogenic factor involved in several central nervous system disorders, such as MS, depression, ischemic brain injury, Alzheimer’s disease, and Parkinson’s disease, Amyotrophic Lateral Sclerosis, and Huntington’s disease (150). Microglial cells, astrocytes, oligodendrocytes, infiltrating myeloid cells are the main reactive cellular components involved in neuroinflammation (151). Microglia are the resident macrophages of the central nervous system, playing a fundamental role in the immune responses in the brain thereby preserving the integrity of neuronal circuits. Remarkably, microglia can exhibit different phenotypes upon different stimuli (152). For example, microglia are classically activated in response to LPS or exposure to pro-inflammatory cytokines (e.g., IFN-γ, TNF-α), displaying a M1 proinflammatory phenotype (153), whereas in response to IL-4 exposure, microglia acquire complex antiinflammatory properties, altering their expression of cytokines, surface markers, phagocytosis capacity, and displaying enhanced potential to induce proliferation of T cells with a regulatory signature (154). Several epigenetic mechanisms, including histone modifications, DNA methylation, and expression of non-coding RNAs (ncRNAs) have been shown to modulate the alteration of microglia phenotypes, allowing these cells to display the ability to adapt and respond to the microenvironment and signal activation (155).

*TET2* has been observed to be upregulated in microglia cells upon TLR-mediated inflammatory stimuli through a NF-κB-dependent pathway. During inflammatory responses, *TET2* was shown to alter transcription of important genes, leading to cellular metabolic reprogramming and expression of different inflammatory mediators. Moreover, in mice models for neuroinflammation and in samples from Alzheimer’s disease patients, *TET2* expression was also shown to be increased. Collectively, these findings suggest that *TET2* is involved in the inflammatory responses in microglia *in vitro* and *in vivo* (156). Contrastingly, *TET2* depletion has also been associated with increased development of neuroinflammation in Alzheimer’s disease. A mouse model for Alzheimer’s disease indicated *TET2* depletion resulted in increased amyloid-β plaque accumulation, microglia overgrowth and proinflammatory cytokine accumulation (e.g., IL-6, IL-1B, and TNF-α) (157).

*TET2* has also been suggested to play a role in microglia proinflammatory activation in Parkinson’s disease. By applying a mice model of inflammation-mediated nigral neurodegeneration, it has been demonstrated that *TET2* loss was accompanied by protection of nigral dopaminergic neurons. Thus, *TET2* inactivation fully prevents nigral dopaminergic neuronal loss induced by previous inflammation. This is of interest as patients with Parkinson´s disease exhibit an epigenetic and transcriptional upregulation of *TET2* (158).

Although astrocytes are essential for protection of neurons, there is growing evidence that astrocytes might also contribute to neuroinflammation when exposed to signals from damaged neurons, proinflammatory microglia and/or damaging insults (e.g., aggregated proteins, and environmental toxicants). Reactive astrocytes acquire the capacity to produce pro-inflammatory cytokines, such as IL-1B, and TNF-α, and ROS. DNA methylation and demethylation have been implicated in the differentiation and function of astrocytes (159, 160). For instance, vitamin C was shown to promote astrocyte differentiation by promoting TET-mediated DNA hydroxymethylation on astrocyte-specific genes (161). The activation of specific pathways including NF-κB and JAK/STAT pathways play an important role in the conversion of reactive astrocytes and in reactive astrogliosis (162).

As DNA demethylation of genes in the JAK-STAT pathway have shown to be important for enhanced activation of STATs for astrocyte differentiation (163) and TETs were shown to affect and be affected by the NF-κB pathway in other cell types (101, 156), it is likely that TETs might also play a role in the neuroinflammatory responses of astrocytes. Furthermore, TET activity seems to be important for generation of oligodendrocytes, which are the myelinating cells of the central nervous system. Oligodendrocyte pathology is therefore evident especially in MS. Oligodendrocytes are generated from oligodendrocyte progenitor cells (164) and all TET proteins (TET1, TET2, and TET3) were shown to play a role in the differentiation process with unique subcellular and temporal expression patterns (165). Moreover, DNA hydroxymethylation catalyzed by TET1 has been shown to be essential for myelin repair in young adults and defective in old mice (166). However, in MS remyelination is often incomplete. Interestingly, in patients with MS *TET2* expression is significantly downregulated in peripheral blood mononuclear cells, which is associated with a decrease of 5-hmdC levels (167). Further studies are still needed to understand the complex role of TET enzymes in the pathophysiology of MS.

## Future Perspectives for Therapeutic Venues

**Figure 6:**
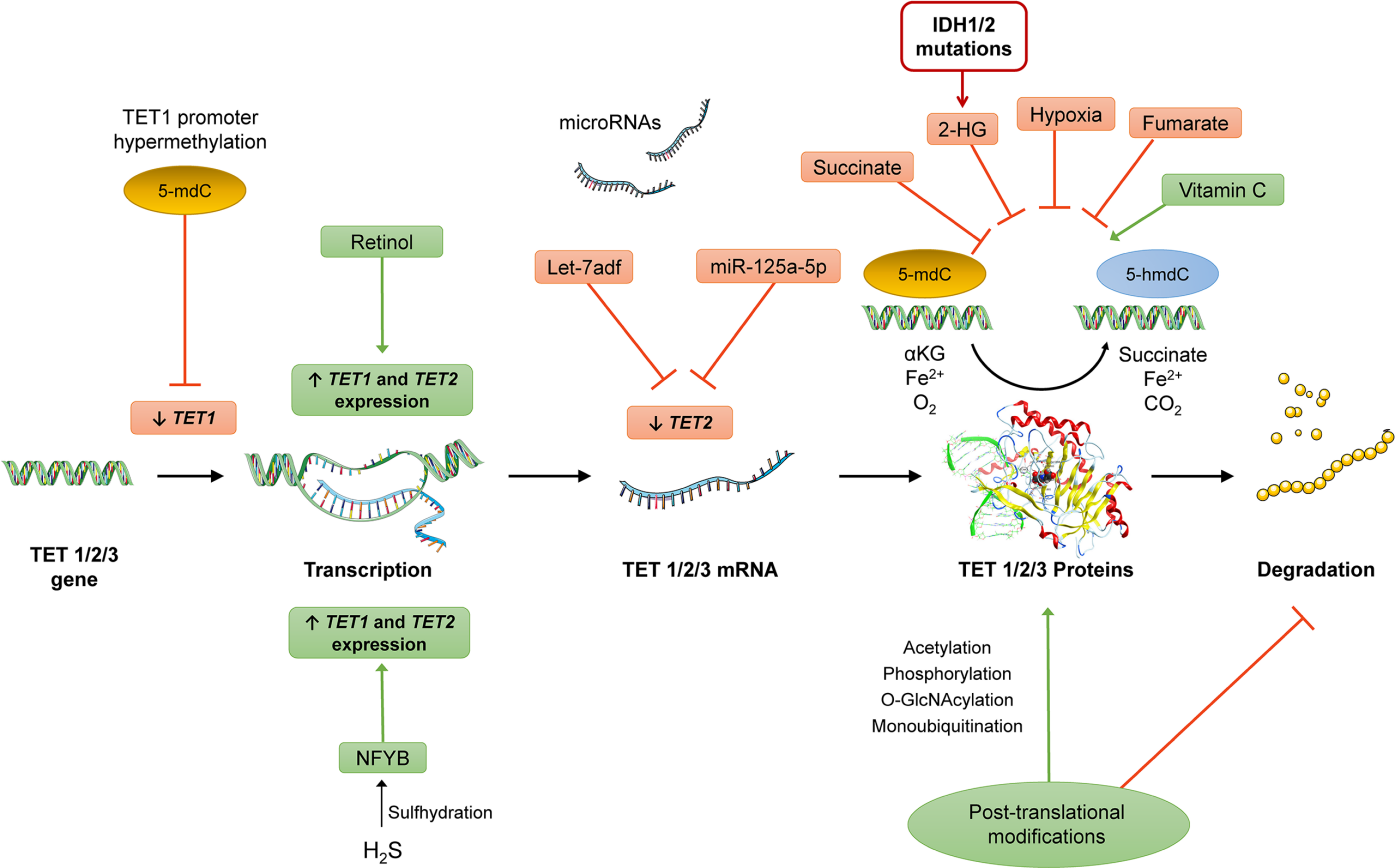
Potential targets for regulation of TET expression at pre-transcriptional (e.g., promoter hypermethylation of TET1l, pro-transcriptional (miRNAs), and post-translational levels (post-translational modifications that can affect TET enzymatic activity and/or stability) and induction/inhibition of TET activity. Different metabolic factors can affect TET enzymatic activity (e.g., 2-HG produced from mutated IDH1/2 enzymes, increased concentrations of succinate, and hypoxia conditions) and other compounds such as vitamin C and retinol were also shown to affect the activity of these enzymes. The nucleic acid images were provided and adapted from Servier Medical Art (smart.servier.com).

The loss of function of *TET*s, through either genetic mutations or catalytic inhibition, has shown a strong causal relationship with multiple malignancies (13, 65, 66). Based on recent discoveries, several new treatment options are now under consideration. Data from a recent meta-analysis has shown that, in spite *TET2* mutations had no significant prognostic value on myelodysplastic syndromes, the response rates to hypomethylating agents were significantly different between patients with and without *TET2* mutations (168). However, in patients with decreased 5-hmdC levels due to elevated miRNA or CXXC expression, TET proteins are still functional. These studies have already demonstrated that *TET* overexpression is able to rescue the phenotype caused by overexpression of *TET*-targeting miRNAs (55, 56). Moreover, the expression of TETs is shown to be regulated on several levels, i.e., at pre-transcriptional, pro-transcriptional (miRNAs), and post-translational levels and, as mentioned before in this review, its activity can be modulated by by re-inducing its activity or enhancing the remaining TET activity by certain compounds like vitamin C (**Figure 6**) (37). For instance, on pre-transcriptional level, *TET1* promoter hypermethylation has been shown to be linked with downregulation of *TET1* and breast cancer metastasis (169). Hydrogen sulfide derived from the metabolism of methionine has shown to lead to sulfhydration of the transcriptional activator nuclear transcription factor Y subunit beta (NFYB), resulting in increased transcription of *TET1* and *TET2* (170). Post-translational modification of TET enzymes, including O-linked-N-acetylglucosamine (O-GlcNAc) Transferase, phosphorylation, Poly-Adenosine Ribosylation (PARylation) and monoubiquitination have shown to significantly affect their enzymatic activity and stability (171). Although *TET* deficiency enhances cell survival and increases cell “stemness”, recent studies discuss the possibility of temporarily inhibiting TET activity in order to enhance immune responses. In chimeric antigen receptor T cells (CAR-T) are efficient therapeutic agent for B lymphocyte malignancies (172).. Here it has been shown, that *TET2* elimination can change the epigenetic map of cells and promote the proliferation of CAR-T cells derived from a single cell clone, thereby promoting remission in leukemia patients and improving the efficacy of immunotherapy (172). Thus, *TET2* deficiency facilitated differentiation and expansion of CD8+ T cells, which could provide protection against tumor and virus infiltration. Non-specific TET inhibitors as 2-hydroxyglutarate should be able to boost antigen-specific responses against tumours by themselves (118). Therefore, there are different potential strategies that could be used for modulating TET function in immune and non-immune cells, potentially furnishing future research with novel therapeutic venues involving TETs to target and ameliorate inflammation-related diseases. It is important to note, however, that these statements are highly exploratory and that the complexity of how TET enzymes regulate immune responses in a cell and tissue type-dependent manner represents a great obstacle for the exploration of these mechanisms for modulating inflammation and in the context inflammation-related diseases. Nevertheless, it appears highly dangerous to misregulate TET activity and 5-hmdC levels indiscriminately. Therefore, more investigations on how to target specific cell types and tissues are still needed. Summarily, intensive further research needs to be done applying different *in vitro* and *in vivo* models to elucidate the mechanisms underlying the complex regulation of the immune system performed by these enzymes and how their modulation might affect ultimately the immune responses.

## Conclusion

Epigenetic regulation plays an important role in modulating immune responses against infection or injury. Besides DNMT-mediated DNA-methylation, the TET enzymes are involved in immune cell development, affecting self-renewal of stem cells and lineage commitment to terminal differentiation. Thus, TETs and DNA-hydroxymethylation are important modulators of immune responses and pathogenesis of inflammatory diseases. Additionally, aberrant DNA-hydroxymethylation plays a key role in dysregulation of HSPC self-renewal and lineage differentiation and can lead to aberrant stem cell function and cellular transformation, leading to both myeloid and lymphoid leukemias. Data demonstrate that TET enzymes regulate a broad range of mechanisms in most of immune cells. Thus, they play an important role in different subset populations of T lymphocytes, affecting their differentiation and function. In addition, they regulate inflammation and autoimmune-related processes in B and plasma cells, as well. Furthermore, TET proteins are involved in the initiation and development of autoimmune diseases like MS and others. Here, the complex role of TET enzymes in the pathophysiology of this is still unclear. In this review, we focused on highlighting the current understanding and emerging concepts in the mechanisms through which TET proteins and their products modulate inflammation in immune and non-immune cells, including also relevant aspects of the regulation of myeloid and lymphoid immune cell development, differentiation, and function. Besides all these fundamental questions, modulating the activity of epigenetic regulating enzymes including TET proteins may be a promising way to alter and to achieve the desired magnitude and direction of immune responses.

## Author Contributions

All authors listed have made a substantial, direct and intellectual contribution to the work, and approved it for publication.

## Funding

We acknowledge support by the Open Access Publication Fund of the Freie Universität Berlin. Dr. Christian Gerecke is funded by Deutsche Forschungsgemeinschaft (DFG - German Research Foundation) grant number GE 3321/2-1.

## Conflict of Interest

The authors declare that the research was conducted in the absence of any commercial or financial relationships that could be construed as a potential conflict of interest.

## Publisher’s Note

All claims expressed in this article are solely those of the authors and do not necessarily represent those of their affiliated organizations, or those of the publisher, the editors and the reviewers. Any product that may be evaluated in this article, or claim that may be made by its manufacturer, is not guaranteed or endorsed by the publisher.

## Abbreviations

2-HG, 2-hydroxyglutarate; αKG, α-ketoglutarate; αKGD, α-ketoglutarate dependent dioxygenases; 5-cadC, 5−carboxyl-2’-deoxycytidine; 5-fdC, 5-formyl-2’-deoxycytidine; 5-hmdC, 5-hydroxymethyl-2’-deoxycytidine; 5-mdC, 5-methyl-2’-deoxycytidine; aa, amino acid; ADAR1, adenosine deaminase acting on RNA 1; AID, activation induced DNA cytosine deaminase; AITL, angioimmunoblastic T cell lymphomas; AML, acute myeloid leukemia; AKT, Akt kinase; BER, base excision repair; CAR, chimeric antigen receptor; CD4/CD8, cluster of differentiation; CEBPα, CCAAT/enhancer-binding protein alpha; CHIP, clonal hematopoiesis of indeterminate potential; CMML, chronic myelomonocytic leukemia; c-myc, cellular myelocytomatosis; CpG, Cytosine Guanine dinucleotide; CXCL1, C-X-C motif chemokine ligand 1; CXXC, cysteine xx cysteine; DC, dendritic cells; DNMTs, DNA-Methyltransferase; FH, fumarate hydratase; FIH, factor inhibiting HIF; FOXP3, forkhead box P3; FTO, fat mass and obesity-associated protein; GATA3, GATA binding protein 3; GC, germinal centers; HDAC, histone deacetylase; HIF, hypoxia inducible factor; HSPC, hematopoietic stem and progenitor cells; IDAX, CXXC finger protein 4; IDH, isocitrate dehydrogenase; IFNB1, interferon beta 1; IFN-γ, interferon gamma; Igκ, Ig light chain; IKBZ, IkappaB zeta; IL, Interleukin; iNKT, invariant natural killer T cell; IRF4, interferon regulatory factor 4; IRF7, interferon regulatory factor 7; JAK, janus kinase; KLF13, Krueppel-like factor 13; let-7adf, miR-let-7a/let-7d/let-7f cluster; LPS, lipopolysaccharide; LSK, Lin^−^Sca-1^+^c-Kit^+^; MDS, myelodysplastic syndromes; MITF, melanocyte inducing transcription factor; MiRNA, microRNA; MLL, mixed-lineage leukemia 1; MNP, myeloproliferative neoplasms; MS, multiple sclerosis; ncRNAs, non-coding RNAs; NF-kB, nuclear factor kappa B; NLRs, NOD-like receptors; NLRP3, NLR family pyrin domain containing 3; NOD, nodulation factors; OGT, *O* ^6^- guanine transferase; OPCs, oligodendrocyte precursor cells; P4H, prolyl-4-hydroxylase; PD-L1, programmed death-ligand 1; PI3K, phosphatidylinositol 3-kinase regulatory subunit alpha; PTEN, phosphatase and tensin homolog; RIG, retinoic acid inducible gene; RLRs, RIG-I-like receptors; ROS, reactive oxygen species; SDH, succinate dehydrogenase; shRNA, short hairpin RNA; Sin3A, SIN3 transcription regulator family member A; SOCS3, suppressor of cytokine signaling; STAT, signal transducer and activator of transcription; TBX21, T-box transcription factor TBX21; Tfh, follicular T helper cells; Th cell, T helper cell; Treg cell, regulatoric T cell; TDG, thymine DNA glycosylase; TET, ten eleven translocation protein; TLRs, toll-like receptors; TNFα, tumour necrosis factor alpha; ZBTB7b, zinc finger and BTB domain-containing protein 7B

## Glossary

**Table:**

2-HG	2-hydroxyglutarate
αKG	α-ketoglutarate
αKGD	α-ketoglutarate dependent dioxygenases
5-cadC	5−carboxyl-2’-deoxycytidine
5-fdC	5-formyl-2’-deoxycytidine
5-hmdC	5-hydroxymethyl-2’-deoxycytidine
5-mdC	5-methyl-2’-deoxycytidine
aa	amino acid
ADAR1	adenosine deaminase acting on RNA 1
AID	activation induced DNA cytosine deaminase
AITL	angioimmunoblastic T cell lymphomas
AML	acute myeloid leukemia
AKT	Akt kinase
BER	base excision repair
CAR	chimeric antigen receptor
CD4/CD8	cluster of differentiation
CEBPα	CCAAT/enhancer-binding protein alpha
CHIP	clonal hematopoiesis of indeterminate potential
CMML	chronic myelomonocytic leukemia
c-myc	cellular myelocytomatosis
CpG	Cytosine Guanine dinucleotide
CXCL1	C-X-C motif chemokine ligand 1
CXXC	cysteine xx cysteine
DC	dendritic cells
DNMTs	DNA-Methyltransferase
FH	fumarate hydratase
FIH	factor inhibiting HIF
FOXP3	forkhead box P3
FTO	fat mass and obesity-associated protein
GATA3	GATA binding protein 3
GC	germinal centers
HDAC	histone deacetylase
HIF	hypoxia inducible factor
HSPC	hematopoietic stem and progenitor cells
IDAX	CXXC finger protein 4
IDH	isocitrate dehydrogenase
IFNB1	interferon beta 1
IFN-γ	interferon gamma
Igκ	Ig light chain
IKBZ	IkappaB zeta
IL	Interleukin
iNKT	invariant natural killer T cell
IRF4	interferon regulatory factor 4
IRF7	interferon regulatory factor 7
JAK	janus kinase
KLF13	Krueppel-like factor 13
let-7adf	miR-let-7a/let-7d/let-7f cluster
LPS	lipopolysaccharide
LSK	Lin^−^Sca-1^+^c-Kit^+^
MDS	myelodysplastic syndromes
MITF	melanocyte inducing transcription factor
MiRNA	microRNA
MLL	mixed-lineage leukemia 1
MNP	myeloproliferative neoplasms
MS	multiple sclerosis
ncRNAs	non-coding RNAs
NF-kB	nuclear factor kappa B
NLRs	NOD-like receptors
NLRP3	NLR family pyrin domain containing 3
NOD	nodulation factors
OGT	*O* ^6^- guanine transferase
OPCs	oligodendrocyte precursor cells
P4H	prolyl-4-hydroxylase
PD-L1	programmed death-ligand 1
PI3K	phosphatidylinositol 3-kinase regulatory subunit alpha
PTEN	phosphatase and tensin homolog
RIG	retinoic acid inducible gene
RLRs	RIG-I-like receptors
ROS	reactive oxygen species
SDH	succinate dehydrogenase
shRNA	short hairpin RNA
Sin3A	SIN3 transcription regulator family member A
SOCS3	suppressor of cytokine signaling
STAT	signal transducer and activator of transcription
TBX21	T-box transcription factor TBX21
Tfh	follicular T helper cells
Th cell	T helper cell
Treg cell	regulatoric T cell
TDG	thymine DNA glycosylase
TET	ten eleven translocation protein
TLRs	toll-like receptors
TNFα	tumour necrosis factor alpha
ZBTB7b	zinc finger and BTB domain-containing protein 7B
